# Effect of calcium chloride injection and freezing prior to wet aging on the meat quality of cull cows

**DOI:** 10.1002/jsfa.70431

**Published:** 2026-01-07

**Authors:** Juliano CC Belmonte, Taynara S Santana, Micheline F Castro, Rosileide V Rohod, Marcelo Vedovatto, Aylpy RD Santos, Henrique J Fernandes, Dalton M Oliveira

**Affiliations:** ^1^ Mato Grosso do Sul State University Aquidauana Brazil; ^2^ Dean Lee Research and Extension Center Louisiana State University Alexandria LA USA

**Keywords:** collagen, meat color, pH, proteolysis, tenderness

## Abstract

**BACKGROUND:**

In Brazil, the slaughter of female cattle, particularly older animals, has increased substantially. However, meat from this category presents limitations in terms of quality, mainly because of its toughness resulting from a high concentration of cross‐links between collagen fibers. The aging process is an effective strategy to improve the quality of meat in this category because it stimulates the activity of cathepsins, which are enzymes responsible for collagen degradation. Calcium chloride (CaCl_2_) injection, in addition to activating calpains, can destabilize lysosomal membranes, releasing cathepsins and enhancing the effects of aging. Additionally, freezing inhibits calpastatins, which are natural inhibitors of calpains, resulting in increased activity of these proteolytic enzymes. Thus, the present study aimed to evaluate the effect of CaCl_2_ injection, in combination with freezing prior to wet aging, on the meat quality of cull cows.

**RESULTS:**

Meat samples injected with CaCl_2_ showed higher b* values. In the samples that were not previously frozen, higher L* values were recorded after 14 days of aging. At 3 days of aging, total collagen content was higher in previously frozen samples, whereas, at 14 days, no effect of freezing was observed.

**CONCLUSION:**

Under the conditions of this study, CaCl_2_ injection, whether combined with freezing or not, minimally affected the quality of aged cull cow meat. Furthermore, prior freezing can be avoided because it negatively impacts meat color. However, when freezing is already part of the industrial process, 14 days of aging has the potential to counteract its adverse effects. © 2026 The Author(s). *Journal of the Science of Food and Agriculture* published by John Wiley & Sons Ltd on behalf of Society of Chemical Industry.

## INTRODUCTION

Brazil holds a prominent position in the global beef production market, being home to the largest commercial cattle herd in the world and leading the global beef export rankings.^1^, ^2^ The Nelore breed is the main beef cattle breed in Brazil, accounting for 80% of the national herd.^3^ The state of Mato Grosso do Sul, located in the Central‐West region of Brazil, stands out in national livestock production because it holds the second largest beef cattle herd in the country and contributes 14% of Brazil's beef exports.^1^

According to IBGE,^4^ 942 568 cows were slaughtered in Mato Grosso do Sul in 2024, and 49% of these females were over 36 months of age. However, meat from cull cows faces challenges related to sensory quality, especially regarding tenderness and color, comprising attributes that are key determinants in consumer purchasing decisions.^5^ It is important to note that meat from older animals, such as females over 36 months of age, contains a greater number of collagen cross‐links, which negatively affects texture and consumer acceptance.^6^, ^7^ In addition, older cattle tend to present darker meat coloration as a result of the increased concentration of myoglobin in the muscle with advancing age,^8^ which may compromise its attractiveness to consumers.

In this context, the use of technologies aimed at improving the meat quality of cull cows should be considered. Among these, the aging process stands out because it promotes significant improvements in tenderness and sensory acceptance through proteolysis driven by endogenous enzymes.^9^ This proteolysis can be evidenced in two stages: within the first 24 h post mortem, calpains act predominantly, favored by the optimal pH during this period.^10^, ^11^ As aging progresses and pH decreases, there is an increase in cathepsin activity,^12^ comprising the enzymes responsible for degrading collagen,^13^ further contributing to meat tenderization. In addition, previous studies have indicated that aging can also contribute to improved meat color.^14^

To enhance these effects, different complementary approaches have been investigated, such as calcium chloride (CaCl_2_) injection and freezing prior to wet aging. The CaCl_2_ injection accelerates the proteolysis process by activating calpains, resulting in improved meat tenderness.^15^ Furthermore, CaCl_2_ injection destabilizes lysosomal membranes, releasing cathepsins and enhancing the effects of aging. Although CaCl_2_ is associated with negative flavor alterations, levels of up to 100 g kg^−1^ do not significantly compromise meat palatability.^16^ Freezing, in turn, not only preserves the product, but also contributes to improved tenderness by inhibiting the activity of calpastatins, which are enzymes that suppress calpain activity.^17^

In the literature, there are no studies that have evaluated the combined effect of CaCl_2_ injection, freezing and wet aging on the meat quality of cull cows over 36 months of age. The hypothesis is that CaCl_2_ injection enhances calpain activity, accelerating aging and improving meat tenderness. Furthermore, CaCl_2_ injection destabilizes lysosomal membranes, releasing cathepsins and enhancing the effects of aging. Additionally, it is considered that prior freezing may intensify the action of these proteolytic enzymes (calpains). Therefore, the present study aimed to evaluate the effects of calcium chloride injection, in combination with freezing prior to wet aging, on the meat quality of cull cows over 36 months of age.

## MATERIALS AND METHODS

### Location

The experiment was conducted at the Carcass Evaluation and Meat Quality Laboratory of the Mato Grosso do Sul State University, Aquidauana Campus (UEMS/UUA), located in the Upper Pantanal region of the Brazilian state of Mato Grosso do Sul.

### Sample and treatments

In total, 144 samples of the longissimus thoracis et lumborum muscle, corresponding to the striploin cut located between the sixth and 12th ribs, were used. The samples were collected from Nelore cows over 36 months of age, classified as having eight permanent incisor teeth according to the Brazilian System for Carcass Classification and Grading. The cows were from the same farm lot and were raised under identical management and conditions. The samples were obtained from a commercial slaughterhouse regulated by the Federal Inspection Service (SIF), following humane slaughter protocols required by Brazilian legislation, in accordance with the Technical Regulation for the Inspection of Animal Products – RISPOA.^18^

**Figure 1:**
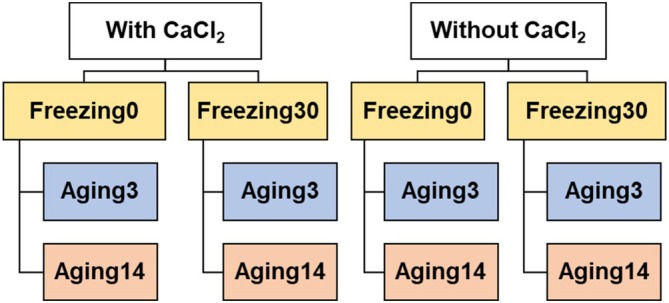
Flowchart representing the adopted experimental design.

The samples were approximately 2.54 cm thick and were allocated in a completely randomized design with a 2 × 2 × 2 factorial arrangement, totaling eight treatments with 18 replicates each. The factors evaluated were: CaCl_2_ injection (with or without), freezing prior to aging (with or without freezing for 30 days) and wet aging time (3 or 14 days) (Fig. 1). Of the 144 samples, 72 received CaCl_2_ injection and 72 did not. In each group (with and without CaCl_2_), 36 samples were subjected to freezing for 30 days prior to aging, and 36 were not frozen. Within each group of 36 samples, 18 were aged for 3 days and 18 for 14 days.

The CaCl_2_ injection was performed using a graduated syringe, injecting the solution into several points of the meat to ensure homogeneous distribution. The solution used had a concentration of 200 mm and was applied in a volume corresponding to 100 g kg^−1^ weight of each sample. After the injection, all of the samples were vacuum‐packed and sent for subsequent procedures according to the experimental treatments. Samples from the non‐frozen treatments were taken directly to the aging step, which lasted for 3 or 14 days. Samples intended for freezing were stored in a freezer at −18 ± 2 °C for 30 days and then subjected to aging for 3 or 14 days, according to the treatment. Aging was carried out in a biochemical oxygen demand type chamber, maintained between 0 and 4 °C.

### Laboratory analysis

At the end of the experimental period, the samples were removed from the packaging for the following laboratory analyses: color (lightness, L*; redness, a*; yellowness, b*; chroma, C*; hue angle, *h*); pigments (metmyoglobin, MMb; deoxymyoglobin, DeoxyMb; oxymyoglobin, O_2_Mb); hydrogen potential (pH); total collagen; cooking loss (CL); and shear force (SF).

For color determination, the meat was placed in plastic trays and kept exposed to air, uncovered, for 30 min to ensure myoglobin oxygenation, and then the values of L*, a*, b*, C* and *h* were measured on the surface of the samples using a spectrophotometer (Minolta CM‐26dg; Minolta Co., Osaka, Japan) calibrated in the CIELAB color system.^19^ The proportions of the chemical forms of myoglobin (pigments) were estimated using the mathematical method of Krzywicki,^20^ based on readings taken with the spectrophotometer, using reflectance curves between 390 and 710 nm at intervals of 10 nm. The pigment content was expressed as g kg^−1^. The pH was determined using a digital pH meter (Mettler, M1120x; Toledo International Inc., Columbus, OH, USA), which was inserted into the geometric center of the samples until the numerical value stabilized.

The total collagen content was determined through the hydroxyproline concentration, which was obtained according to the methodology described by Neuman and Logan,^21^ with modifications proposed by Bergman and Loxley.^22^ For this, 4 g of meat sample was ground using a shaker and then mixed with 30 mL of sulfuric acid solution for hydrolysis, which was carried out in an oven at 105 °C for 16 h. The hot hydrolyzed content was transferred to a 500‐mL volumetric flask, and the volume was completed with distilled water. After cooling the mixture to room temperature, the content was filtered. A 500‐μL aliquot of the filtrate was transferred to a 10‐mL test tube containing 1500 μL of distilled water. Then, 1 mL of oxidizing solution, composed of 200 g kg^−1^ chloramine‐T stock solution and 800 g kg^−1^ citrate buffer, was added. The mixture was homogenized using a vortex shaker and left to stand at room temperature for 20 min. Subsequently, 1 mL of Ehrlich's reagent (prepared with 10 g of *p*‐dimethylaminobenzaldehyde dissolved in 35 mL of 600 g kg^−1^ perchloric acid and diluted in 65 mL of isopropanol) was added. The tubes were again vortexed, wrapped in aluminum foil, and incubated in a water bath at 60 °C for 15 min. After this period, they were cooled in running water for 3 min. The samples were removed from the tubes, placed in cuvettes, and read in a spectrophotometer at an absorbance of 558 nm, using the blank as a control. The obtained hydroxyproline values were multiplied by 7.14 to estimate the total collagen content.^23^

For the determination of CL, the meat samples were weighed and placed in an electric oven preheated to 180 °C until the center of the sample reached 71 °C. A digital thermometer (KP‐8007; KNUP, China) was used to monitor the temperature. After cooking, the samples were weighed again, and the weight loss was calculated as the ratio between the difference in weight and the initial weight, expressed as g kg^−1^.

Once the samples were weighed to determine the CL, three cylinders were extracted from each meat sample, parallel to the orientation of the muscle fibers, using a standardized coring device with a diameter of 1.27 cm. The cylindrical subsamples were then cut transversely using a V‐shaped Warner‐Bratzler blade with a 60° angle and a fixed speed of 3.3 mm s^−1^, attached to a texture analyzer (Brookfield CT; AMETEK Brookfield, Middleboro, MA, USA). The peak SF was measured in Newtons and the averages were obtained from the values of the three cylinders from each sample.

### Statistical analysis

All analyses were performed using the sample as the experimental unit. The effect of the treatments was evaluated using the MIXED procedure of SAS, version 9.4 (SAS Institute Inc., Cary, NC, USA), and CaCl_2_ injection, freezing and aging time were included as fixed effects, along with all possible interactions among these factors. Means were separated using the PDIFF function, and all results were reported as LSMEANS followed by the standard error of the mean (SEM). *P* < 0.05 was considered statistically significant.

## RESULTS

Table 1 presents the data related to the parameters evaluated as a function of prior freezing, CaCl_2_ injection and maturation time.

**Table 1:** Effect of CaCl_2_ injection and freezing prior to 3‐ and 4‐day wet aging on the meat quality of cull cows

	With CaCl_2_				Without CaCl_2_				SEM	*P‐*value						
	Aging3		Aging14		Aging3		Aging14			Ca	A	F	Ca × A	Ca × F	A × F	Ca × A × F
Items	Freezing0	Freezing30	Freezing0	Freezing30	Freezing0	Freezing30	Freezing0	Freezing30								
L*	35.2	36.0	37.2	36.9	34.2	36.0	37.1	36.2	0.64	0.31	< 0.01	0.45	0.92	0.90	0.03	0.35
a*	16.7	11.8	15.1	15.3	15.9	11.8	15.3	14.5	0.42	0.24	< 0.01	< 0.01	0.95	0.80	< 0.01	0.14
b*	13.7	12.4	13.5	13.8	12.7	11.5	13.7	13.3	0.38	0.03	< 0.01	0.01	0.15	0.63	0.01	0.48
C*	21.7	17.2	19.6	20.7	20.5	16.6	20.6	19.7	0.53	0.22	< 0.01	< 0.01	0.23	0.36	< 0.01	0.11
*h*	39.5	46.1	41.8	42.0	38.7	44.1	41.9	42.5	0.92	0.41	0.90	< 0.01	0.18	0.75	< 0.01	0.52
pH	6.5	5.7	5.4	5.4	6.6	5.8	5.4	5.4	0.02	< 0.01	< 0.01	< 0.01	0.01	0.92	< 0.01	0.98
TC (g kg^−1^)	2.6	4.2	3.4	4.5	2.8	3.1	4.5	3.5	0.09	0.11	< 0.01	< 0.01	0.09	< 0.01	< 0.01	0.13
CL (g kg^−1^)	309.0	323.0	368.0	320.4	272.0	323.0	292.0	337.5	7.19	0.03	0.07	0.14	0.82	0.01	0.12	0.63
SF (N)	25.3	31.1	30.9	28.9	27.9	31.1	31.0	30.6	1.50	0.30	0.15	0.11	0.83	0.81	< 0.01	0.33
MMb (g kg^−1^)	327.0	385.0	315.4	315.4	334.2	371.0	287.3	307.5	4.10	0.13	< 0.01	< 0.01	0.31	0.97	0.01	0.15
DeoxyMb (g kg^−1^)	44.7	61.9	44.6	43.2	67.3	79.2	55.4	80.9	2.95	< 0.01	0.20	0.01	0.70	0.33	0.82	0.15
O_2_Mb (g kg^−1^)	628.3	553.0	640.0	641.3	598.4	550.0	657.2	611.5	4.57	0.14	< 0.01	< 0.01	0.50	0.50	0.01	0.13

*Note*: With CaCl_2_, with CaCl_2_ injection; Without CaCl_2_, without CaCl_2_ injection; Aging3, aging for 3 days; Aging14, aging for 14 days; Freezing0, without freezing; Freezing30, freezing for 30 days;

Abbreviations: L*, lightness; a*, redness; b*, yellowness; C*, chroma; *h*, hue angle; pH, hydrogen potential; TC, total collagen; CL, cooking loss; SF, shear force; MMb, metmyoglobin; DeoxyMb, deoxymyoglobin; O_2_Mb, oxymyoglobin; SEM, standard error of the mean; Ca, effect of the CaCl_2_ injection; A, effect of the aging time; F, effect of the freezing; Ca × A, effect of the interaction between CaCl_2_ injection and aging time; Ca × F, effect of the interaction between CaCl_2_ and freezing; A × F, effect of the interaction between aging time and freezing; Ca × A × F, effect of the interaction between CaCl_2_ injection, aging time, and freezing.

### Individual effects

**Figure 2:**
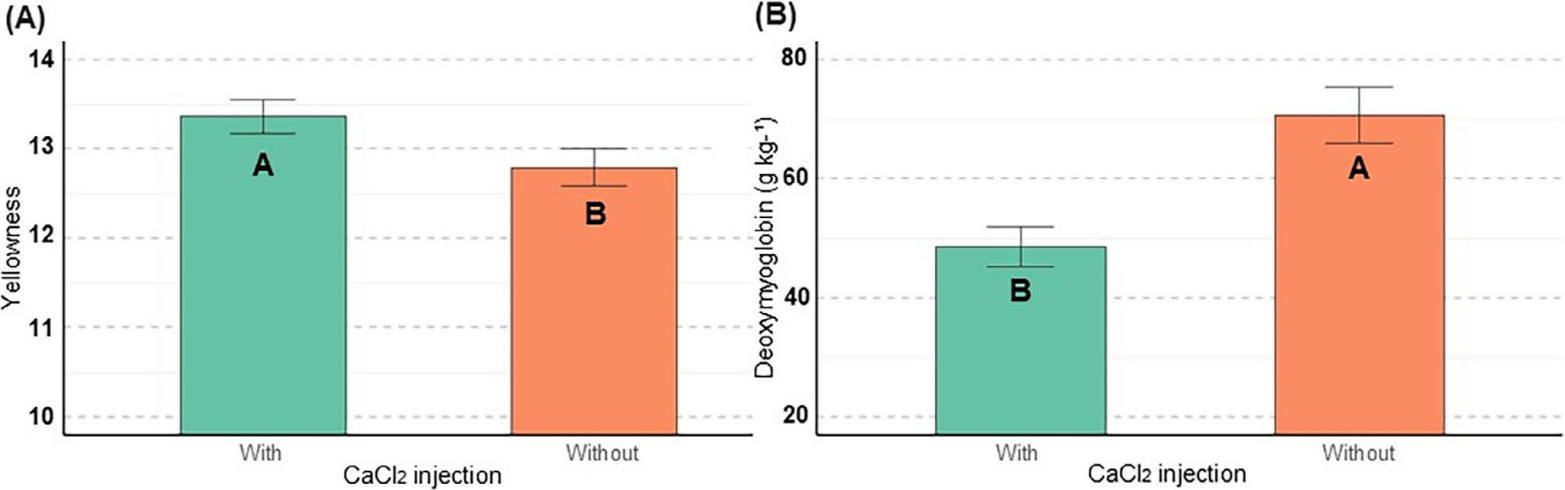
Effect of CaCl_2_ injection on yellowness (A) and deoxymyoglobin (B) values in the meat of cull cows; different letters indicate significant differences.

**Figure 3:**
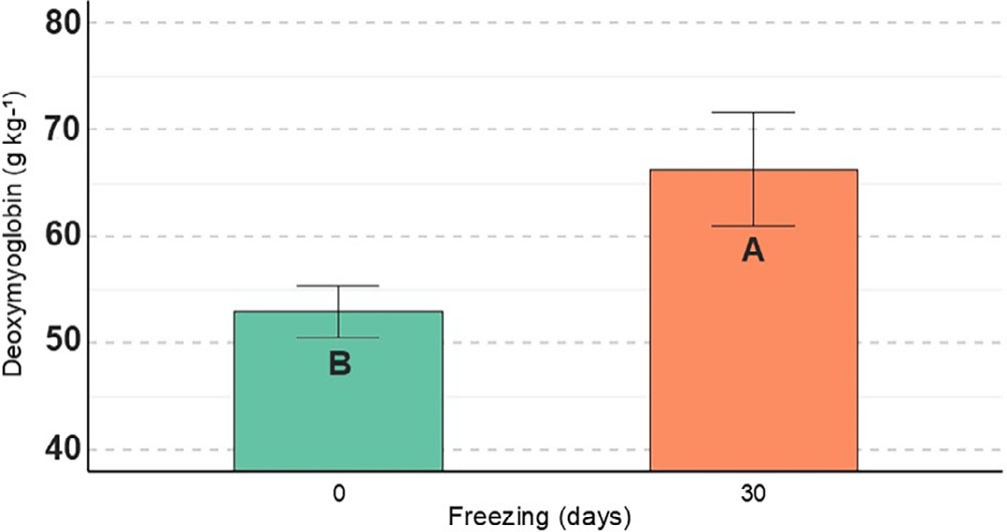
Effect of freezing prior to aging on deoxymyoglobin values in meat of cull cows; different letters indicate significant differences.

Meat samples injected with CaCl_2_ showed higher b* values (*P* = 0.03) (Fig. 2(A)). In addition, CaCl_2_ injection and prior freezing affected the DeoxyMb content of the meat samples. Meat samples injected with CaCl_2_ had a lower concentration of DeoxyMb (*P* < 0.01) (Fig. 2(B)), whereas samples subjected to prior freezing showed a higher concentration (*P* = 0.01) (Fig. 3).

### Effect of the interaction between CaCl_2_ injection and aging time

**Figure 4:**
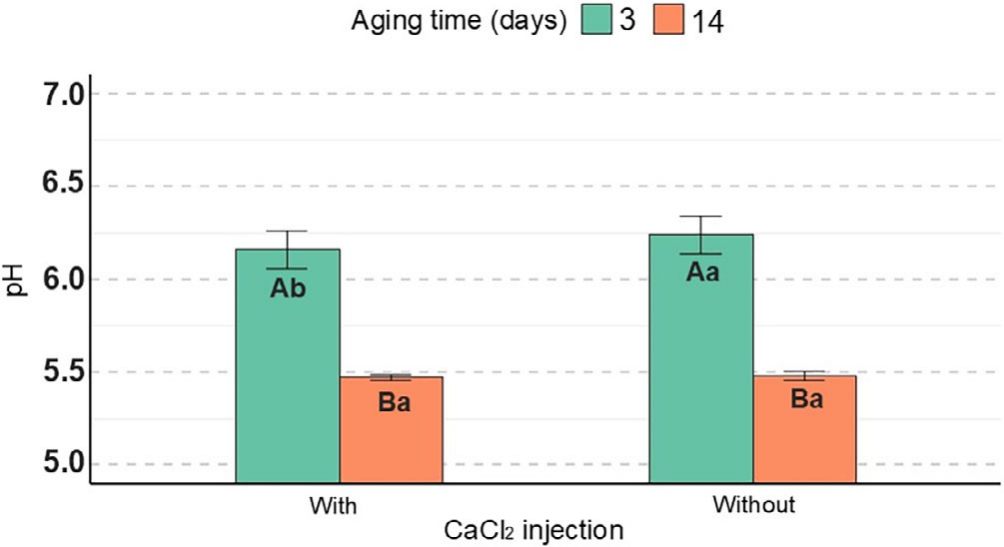
Effect of CaCl_2_ injection and aging time on pH value in meat of cull cows; uppercase letters indicate differences as a result of aging time, whereas lowercase letters indicate differences as a result of CaCl_2_ injection.

An interaction between aging time and CaCl_2_ injection was observed for the pH of the meat samples (*P* = 0.01). In all of the samples (with and without CaCl_2_ injection), the 14‐day aging period resulted in lower pH compared to samples aged for 3 days (Fig. 4). At 3 days of aging, CaCl_2_ injection led to a reduction in pH. However, at 14 days, no effects of CaCl_2_ injection were observed for this parameter.

### Effect of the interaction between CaCl_2_ injection and freezing

**Figure 5:**
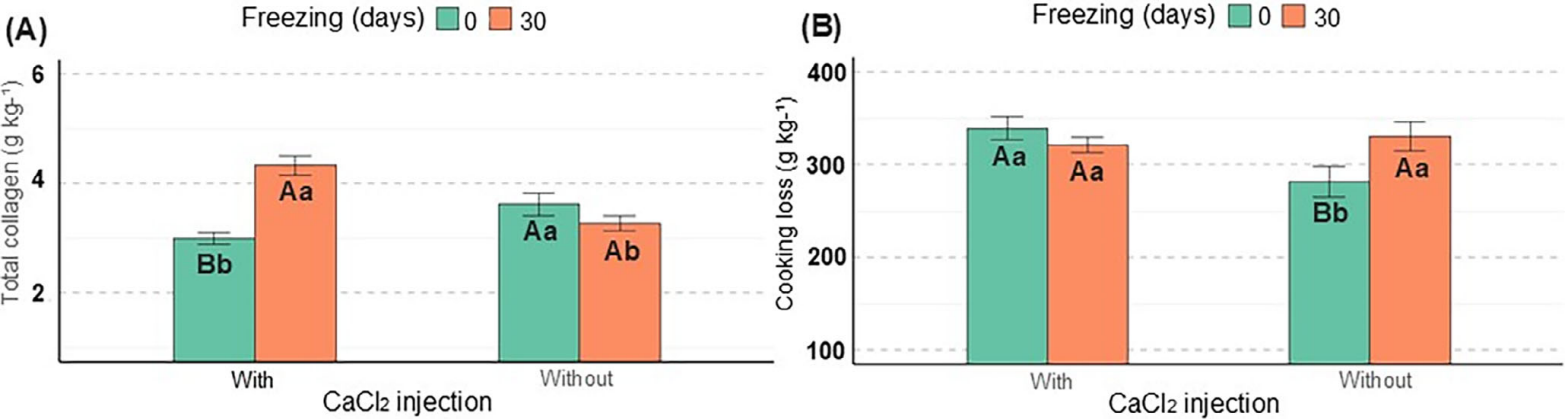
Effect of CaCl_2_ injection and freezing on total collagen (A) and cooking loss (B) values in meat of cull cows; uppercase letters indicate differences as a result of freezingwhereas lowercase letters indicate differences as a result of CaCl_2_ injection.

An interaction between CaCl_2_ injection and prior freezing was observed for total collagen and CL values (*P* < 0.05). In the meats that were previously frozen, CaCl_2_ injection increased the total collagen concentration (Fig. 5(A)). In the non‐frozen meats, the opposite was observed, in that CaCl_2_ injection decreased total collagen. Furthermore, in the samples that received CaCl_2_ injection, freezing increased total collagen, whereas, in the samples that did not receive CaCl_2_ injection, no freezing effect was observed. In samples that did not receive CaCl_2_ injection, freezing increased CL values (Fig. 5(B)). Conversely, in samples where CaCl_2_ was not injected, no freezing effect was observed. The effects of CaCl_2_ injection were only detected in non‐frozen samples, where CaCl_2_ injection increased CL values.

### Effect of the interaction between freezing and aging time

**Figure 6:**
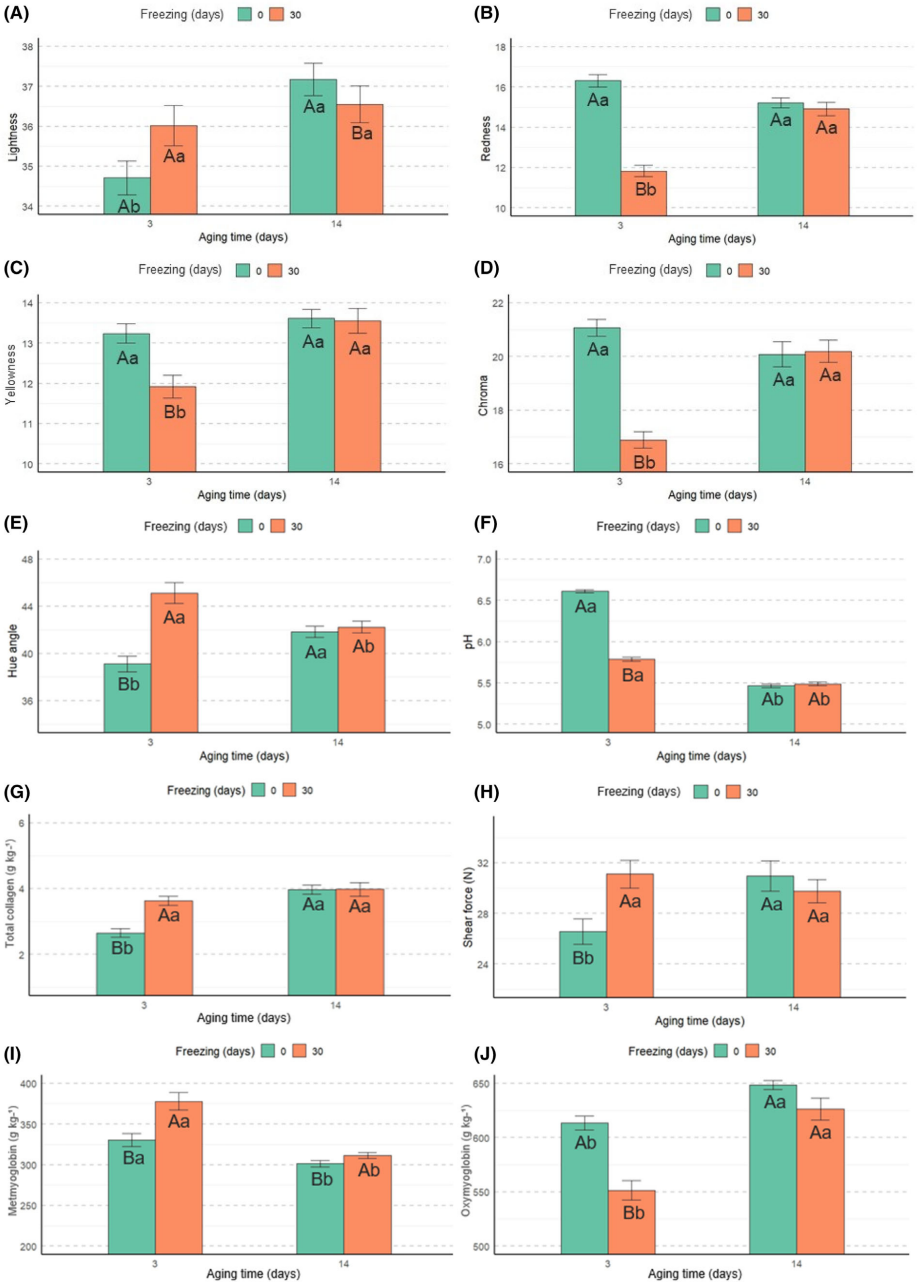
Effect of freezing and aging time on the meat quality parameters (lightness, (A); redness, (B); yellowness, (C); chroma, (D); hue angle, (E); pH, (F); total collagen, (G); shear force, (H); metmyoglobin, (I); oxymyoglobin, (J)) of cull cows; uppercase letters indicate differences as a result of freezing, whereas lowercase letters indicate differences as a result of aging time.

There was an interaction (*P* < 0.05) between freezing and aging time on the values of L*, a*, b*, C*, *h*, pH, total collagen, SF, MMb and O_2_Mb in the meat samples. In samples that were not previously frozen, higher L* values were recorded after 14 days of aging compared to those aged for 3 days (Fig. 6(A)). However, in samples that were subjected to prior freezing, L* values did not vary according to aging time. It was observed that after 14 days of aging, frozen samples showed lower L* values compared to non‐frozen samples.

The effect of aging time on a* values was observed only in meat samples that underwent freezing, where 14 days of aging resulted in higher a* values (Fig. 6(B)). Regarding the effects of freezing, in samples aged for 3 days, the absence of prior freezing resulted in higher a* values. After 14 days of aging, there was no effect of prior freezing on a* values. According to Fig. 6(C), aging time had no influence on b* values in samples that were not previously frozen. On the other hand, in previously frozen samples, 14 days of aging resulted in higher b* values. Additionally, it was found that after 3 days of aging, b* values were higher in samples that had not been subjected to freezing.

Aging time affected only the C* values of meat samples that were subjected to prior freezing (Fig. 6(D)), where 3 days of aging resulted in lower values for this parameter. Additionally, the effect of freezing was only observed at 3 days of aging, with previously frozen samples presenting lower C* values compared to those that were not frozen. Regarding *h*, in previously frozen meat samples, 14 days of aging resulted in lower values for this parameter (Fig. 6(E)). In non‐frozen samples, the opposite was observed. Furthermore, at 3 days of aging, a freezing effect was observed for this parameter, with prior freezing resulting in higher h values.

In all of the samples (frozen or not), 14 days of aging resulted in lower pH values (Fig. 6(F)). After 3 days of aging, the pH of the non‐frozen samples was higher compared to the previously frozen samples. Regarding total collagen content, lower values were found in samples aged for 3 days (Fig. 6(G)). Moreover, at 3 days of aging, total collagen content was higher in previously frozen samples, whereas, at 14 days, no effect of freezing was observed.

The SF values were similar between aging times in the meat samples subjected to freezing (Fig. 6(H)). On the other hand, in the non‐frozen samples, lower SF values were obtained in the samples aged for 3 days. Regarding the effects of freezing, it was observed that, in samples aged for 3 days, freezing increased SF values, whereas, in meats aged for 14 days, freezing had no significant effect. Regarding the MMb content, the 14‐day aging time resulted in lower values of this parameter, both in the samples previously frozen and in those that were not frozen (Fig. 6(I)). After 3 days of aging, higher MMb values were observed in the frozen samples, whereas, after 14 days of aging, the opposite trend was found.

There was an interaction between prior freezing and aging time on the O_2_Mb values (*P* = 0.01) (Fig. 6(J)). In both frozen and non‐frozen meat samples, the 14‐day aging time resulted in an increase in O_2_Mb values. Furthermore, it was observed that, with 3 days of aging, freezing decreased the concentration of this parameter. On the other hand, at 14 days, no effect of freezing was observed.

## DISCUSSION

The present study was based on the hypothesis that the benefits of aging on the meat quality of cull cows would be enhanced by prior freezing, and especially by the application of CaCl_2_. However, the findings indicated that CaCl_2_ injection had a limited effect compared to the other factors evaluated, and thus most of the results were the result of an interaction between freezing and aging time.

In meats aged for 3 days, freezing causes an increase in L* values. In meats aged for 14 days, however, freezing leads to a slight decrease in L* values because longer aging periods significantly increased the L* of meats that were not previously frozen. Similar results were observed by Aroeira *et al*.,^14^ who found that meat samples frozen before aging exhibited lower L* values, resulting in a darker appearance. According to Aroeira *et al*.,^14^ this behavior is a result of the increased concentration of solutes in the intracellular medium caused by freezing, which reduces light reflection and gives the meat a darker color. It is important to note that freezing, especially when prolonged, intensifies lipid oxidation and significantly increases MMb levels.^24^ In the present study, conducting an analysis of lipid oxidation could confirm this hypothesis. However, future studies focused on investigating the effects of freezing on lipid oxidation are strongly recommended.

This explanation aligns with the findings of the present study, in which non‐frozen meat showed slightly lower MMb levels. Lanari *et al*.^25^ reported higher MMb concentrations in meat samples subjected to freezing. According to Abdallah *et al*.,^26^ the enzymatic system responsible for reducing MMb is negatively affected by freezing, which explains the accumulation of MMb and the lower concentration of O_2_Mb in previously frozen meat samples. Moreover, in the frozen meat samples, DeoxyMb levels were higher than in non‐frozen samples. This result supports the hypothesis that freezing increases solute concentration in meat, leading to elevated pigment levels.

It is assumed that the higher MMb content in frozen meat samples was responsible for the reduction in a* and C* values, as well as for the increase in *h* values. The lower b* values observed in frozen samples aged for 3 days can be partially explained by the MMb content. Negative effects of freezing on b* values have been reported in other studies.^27^, ^28^

Although freezing increased MMb values and reduced O_2_Mb values, when a 14‐day aging period was applied, this effect was less pronounced, suggesting that extending the aging time may improve the color of previously frozen meats. Regarding the effects of aging time on L* values, in non‐frozen samples, an increase in L* values was observed as the aging period increased. Cho *et al*.^29^ also reported increased L* values in beef as aging days progressed. Similarly, Aroeira *et al*.^14^ and Gómez *et al*.^30^ found higher L* values in meat subjected to longer aging periods. Several previous studies in the literature^30^, ^31^, ^32^, ^33^ have suggested that the increase in L* values observed with longer aging times is associated with reduced mitochondrial respiratory activity, which enhances the oxygenation of the myoglobin molecule, resulting in greater O_2_Mb formation.

In the present study, the lower MMb content as a result of an increased aging time also explains the rise in L* values. Moreover, after 14 days of aging, an increase in O_2_Mb values was observed, which negatively correlates with MMb levels because both are associated with the reduced and oxidized states of the iron atom bound to the myoglobin molecule, respectively. Thus, considering that O_2_Mb is associated with a more attractive meat color, increasing the aging time may be a strategy to improve the color of meats subjected to freezing.

The decrease in pH with increasing aging time has been reported in other studies.^34^, ^35^, ^36^ According to Blixt and Borch,^37^ aging vacuum‐packed meats results in the accumulation of lactic acid produced by lactic bacteria naturally present in the meat, leading to a pH decline. A microbiological analysis could support these findings; however, in the present study, such an analysis was not performed, with this analysis being recommended for future experiments. In the present study, frozen meat showed a lower pH value after 3 days of aging. Leygonie *et al*.^24^ report that freezing increases protein denaturation and promotes the release of H^+^ ions into the medium, resulting in a pH decline. In a recent study, Guimarães *et al*.^38^ also observed lower pH values in beef samples subjected to freezing prior to aging.

With 3 days of aging, non‐frozen meat samples showed lower total collagen content compared to frozen samples, which helps explain the lower SF observed. The increase in total collagen concentration in meats subjected to freezing likely occurred because of water loss caused by freezing, which elevated the concentration of solutes. Although we did not assess weight loss as a result of exudation, recent studies^38^, ^39^ have shown that ice crystals formed during freezing damage the structural integrity of cell membranes, increasing the flow of water into the extracellular space. This explanation accounts for the higher CL values observed in the samples that underwent freezing.

It was expected that the longer aging period (14 days) would result in lower SF values, especially in the samples that had been frozen, because freezing enhances *postmortem* proteolysis.^39^, ^40^ In a recent study, Guimarães *et al*.^38^ observed a decrease in beef SF values as aging time increased. Similarly, in a study on beef stored in different packaging systems, Santos‐Donado *et al*.^41^ observed that increasing the aging time enhanced the tenderness of vacuum‐packaged meat from Nelore cows. However, the values reported are higher than those observed in the present study. The lower SF value found in non‐frozen meat aged for 3 days suggests that, under this combination of factors, desirable tenderness can be achieved with a shorter aging period.

It is important to highlight that, in the present study, the effects of prior freezing were more evident in samples subjected to a short aging period (3 days), indicating that prolonged aging has a predominant impact on the meat quality of cull cows. In this regard, when longer aging periods, such as 14 days, are applied, freezing becomes unnecessary because it does not provide additional improvements in meat quality compared to non‐frozen meat. Nevertheless, if freezing is required in the industry, a 14‐day aging period can be adopted because it offsets the negative effects of freezing on most quality parameters. In the literature, aging periods of 14 days have been commonly adopted in studies with cull cow meat because this duration is sufficient to promote tenderization and improve the sensory attributes of the product.^42^, ^43^ A sensory analysis could support this hypothesis and is therefore recommended for future investigations.

It was expected that CaCl_2_ injection would enhance the effects of aging by reducing SF values, especially after 3 days of aging. According to Gomide *et al*.,^44^ the Ca^2+^ ion not only stimulates calpain activity during the post‐mortem period, but also alters the ionic strength in the meat, affecting lysosomal membrane stability, which results in greater cathepsin release, enzymes responsible for increasing collagen solubility. This, combined with the pH decline, should have promoted more extensive proteolysis and consequently lower SF values, which was not observed in this study. The increase in CL in non‐frozen meat samples suggests that CaCl_2_ injection enhanced muscle fiber fragmentation; however, this fragmentation was not sufficient to affect SF.

The lack of effect of CaCl_2_ on tenderness may be related to the aging durations used. It is important to highlight that longer aging periods reduce meat pH, which in turn decreases calpain activity.^11^ Therefore, the effects of CaCl_2_ injection are more evident within the first 24 h post mortem.^44^ Analyzing CaCl_2_‐injected meat within the first 24 h post mortem could confirm this theory.

It is noteworthy that, in the present study, all treatments resulted in low SF values, remaining within the threshold of up to 48 N, which classifies the meat as tender.^45^ In a study conducted by Bunmee *et al*.,^15^ which evaluated the combined effects of aging and CaCl_2_ injection on the meat quality of cull cows, a reduction in SF values was observed as a result of these factors. However, the values reported in that study were relatively high and exceeded the 48 N threshold. Therefore, although the combined effects of these factors were limited in the present study, their impact on improving tenderness was achieved.

Regarding the effects of CaCl_2_ on DeoxyMb content and b* values, the literature presents few studies linking the injection of this additive to changes in meat color. Therefore, our explanation on this aspect is limited. However, Wheeler *et al*.^16^ explain that CaCl_2_ may promote pigment oxidation, which can lead to an increase in b* values and a decrease in DeoxyMb content. In future studies, an analysis of lipid oxidation is strongly encouraged. In non‐frozen samples, CaCl_2_ reduced total collagen content but had no effect on meat tenderness.

In the present study, we hypothesized that the positive effects of aging on the meat quality of cull cows would be enhanced by prior freezing and, especially, by CaCl_2_ injection. However, the results showed that CaCl_2_ injection had minimal impact on meat quality compared to the other factors evaluated. This is likely because all of the samples in this study were subjected to either 3 or 14 days of aging, which led to a pH decline and reduced calpain activity, with such enzymes being calcium‐dependent. It is possible that, under these aging durations, other mechanisms contributed to the tenderization of meat samples that did not receive CaCl_2_ injection, creating a compensatory effect and thus explaining the similar outcomes. As a result, most of the findings were based on an interaction between freezing and aging.

Overall, CaCl_2_ injection does not appear to be necessary when using 3 or 14 days of aging. In non‐frozen samples, CaCl_2_ reduced total collagen content but had no effect on meat tenderness. Under short aging conditions (3 days), freezing should be avoided because of its detrimental effects on meat quality. By contrast, in previously frozen meats, a 14‐day aging period shows great potential to improve quality traits. Regarding the evaluation of CaCl_2_, further research is recommended, particularly including day 0 of aging.

## AUTHOR CONTRIBUTIONS

JCCB was responsible for conceptualization. JCCB, TSS, MFC, RVR, HJF and DMO were responsible for investigation. JCCB, TSS, MFC, RVR and MV were responsible for data curation. JCCB and MV were responsible for software. TSS, MFC, RVR and MV were responsible for methodology. MV and DMO were responsible for conceptualization. MV, ARDS and DMO were responsible for formal analysis. ARDS and DMO were responsible for resources. ARDS and DMO were responsible for validation. ARDS was responsible for writing the original manuscript. ARDS, HJF and DMO were responsible for reviewing and editing. HJF and DMO were responsible for project administration. HJF and DMO were responsible for supervision.

## FUNDING

No funding was received for this work.

## CONFLICTS OF INTEREST

The authors declare that they have no conflicts of interest.

## Data Availability

The data that support the findings of this study are available from the corresponding author upon reasonable request.
